# Subcongenic analysis of *tabw2* obesity QTL on mouse chromosome 6

**DOI:** 10.1186/1471-2156-13-81

**Published:** 2012-10-01

**Authors:** Taryn P Stewart, Xia Mao, Maha N Aqqad, Deon Uffort, Kristy D Dillon, Arnold M Saxton, Jung Han Kim

**Affiliations:** 1 Department of Pharmacology, Physiology and Toxicology, Joan C. Edwards School of Medicine, Marshall University, 1700 3rd Ave. BBSC #435K, Huntington, WV, 25755, USA; 2 Department of Nutrition, The University of Tennessee, Knoxville, TN, 37996, USA; 3 Department of Animal Science, The University of Tennessee, Knoxville, TN, 37996, USA

## Abstract

**Background:**

We previously established a congenic mouse strain with TALLYHO/Jng (TH) donor segment on chromosome 6 in a C57BL/6 (B6) background that harbors an obesity quantitative trait locus, *tabw2*. The B6.TH-*tabw2* congenic mice developed increased adiposity that became exacerbated upon feeding a high fat-high sucrose (HFS) diet. To fine map the *tabw2*, in this study we generated and characterized subcongenic lines with smaller TH donor segments.

**Results:**

We fixed four subcongenic lines, with maximum size of donor segment retained in the lines ranging from 10.8 – 92.5 Mb. For mapping, all the subcongenic mice, along with B6.TH-*tabw2* congenic and B6-homozygous control mice were fed either chow or HFS diets, and their post-mortem fat pads were weighed. Mice were also characterized for energy expenditure, respiratory exchange ratio, locomotor activity, and food intake. As previously reported, B6.TH-*tabw2* congenic mice showed a significantly larger fat mass than controls on both diets. On chow, a subcongenic line retaining the distal region of the TH donor congenic interval exhibited significantly larger fat mass than B6-homozygous controls, and comparable that to B6.TH-*tabw2* congenic mice. Two nested subcongenic lines within that region suggested that the effect of *tabw2* on obesity could be attributed to at least two subloci. On HFS diets, on the other hand, all the subcongenic mice had significantly larger fat mass than controls without genotype differences, but none of them had fat mass as large as the original congenic mice. This possibly implicates that further genetic complexity involves in the effect of *tabw2* on diet-induced obesity. Significantly reduced locomotor activity was exhibited in B6.TH-*tabw2* congenic and subcongenic mice compared to controls when animals were fed HFS diets. B6.TH-*tabw2* congenic mice, but not subcongenic mice, also had significantly increased food intake on HFS diets.

**Conclusions:**

It appears that at least two subloci explaining the *tabw2* effect under chow feeding map to the distal region of the congenic interval, whereas the diet-induced obesity mediated by *tabw2* is attributed to more complex genetic mechanism.

## Background

Obesity is characterized by excessive storage of fat in adipose tissue [1]. The etiology of human obesity involves genetic predisposition from multiple genes and nongenetic risk factors such as sedentary lifestyle and energy-dense diets [2-5]. Closely resembling this disease in humans, polygenic mouse model of obesity with natural variants offers a valuable alternative for studying genetic architecture of obesity [6,7]. One powerful application available with mouse models is construction of congenic mice. Congenic mouse strains are virtually identical except for a small donor chromosomal segment harboring a gene of interest [8,9]. Therefore, congenic mice allow the multiple genes underlying polygenic obesity to be dissected into individual genes for further study.

TALLYHO/Jng (TH) mice are a polygenic inbred model of type 2 diabetes and obesity [10]. Previously, we have established a congenic mouse strain that harbors a TH-derived chromosome 6 genomic segment containing an obesity quantitative trait locus (QTL), *tabw2* (TALLYHO associated body weight 2), on a B6 background [11]. This B6.TH-*tabw2* congenic strain develops increased adiposity that becomes exacerbated upon feeding high fat-high sucrose (HFS) diets. In this study, we generated and characterized subcongenic lines of mice with smaller TH donor segments to fine map the *tabw2* QTL. Unlike conventional linkage mapping, such as with F2 cross, multiple mice with the same recombination can be analyzed from subcongenic lines, resulting in more accurate estimation of the phenotype conferred by the QTL. This subcongenic approach has been successfully used for refining the location of QTLs and identification of susceptibility genes for diabetes and obesity [12-14].

## Results

### Generation of subcongenic lines of mice for *tabw2* QTL on chromosome 6

**Figure 1:**
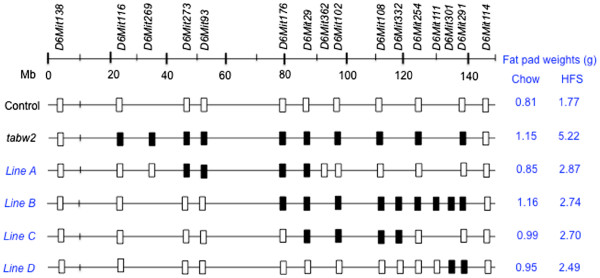
**B6.TH-*tabw2 *congenic and subcongenic intervals at the *tabw2 *locus on mouse chromosome 6.** SSLP primers shown at the top were used to allelotype the congenic interval. The open and filled boxes represent a B6 and TH allele, respectively. Mean fat pad weights (g) are listed for each group under two diets for comparison (detailed phenotypic data are presented in Table 1).

To fine map the *tabw2* QTL, subcongenic lines of mice with different segments of the TH donor congenic interval were generated from B6.TH-*tabw2* congenic mice (Figure 1). These subcongenic lines captured the full congenic region, except the end-proximal portion of ~20-Mb. The maximal TH interval retained in the lines, marked as the most proximal and distal primers with known B6 homozygous genotypes for a given donor segment, ranged from 10.8 (line D) – 92.5 (line B) Mb. The minimal TH interval retained in the lines, marked as the most proximal and distal primers with known TH homozygous genotypes for a given donor segment, ranged from 1.4 (line D) – 57.2 (line B) Mb. Alleotype of the genomic region between maximal and minimal interval primers is unknown.

### Mapping of the adiposity trait using subcongenic mice

For subcongenic mapping, male B6.TH-*tabw2* congenic, subcongenic, and B6-homozygous control mice were fed either chow or HFS diets, and body and fat pad weights were measured. As previously reported [11], B6.TH-*tabw2* congenic mice showed a significantly larger fat pad weight than B6-homozygous control mice on both diets (Table 1). Therefore, we used fat pad weight as the phenotypic trait for fine mapping.

**Table 1:** **Body and fat pad weights of B6.TH-*tabw2 *congenic, subcongenic, and B6-homozygous control mice on chow and high fat-high sucrose diets (HFS) (males)**

	**TH genome retained (Mb)**		**Body weight (g)**		**Fat pad weight (g)**	
**Group**	**Maximum**	**Minimum**	**Chow**	**HFS**	**Chow**	**HFS**
Control	0	0	28.7 ± 0.4^a^ (n = 15)	29.6 ± 0.7^d^ (n = 17)	0.81 ± 0.06^c^ (n = 15)	1.77 ± 0.27^c^ (n = 17)
*tabw2*	140.1	112.3	28.4 ± 0.5^ab^ (n = 13)	36.9 ± 1.1^a^ (n = 7)	1.15 ± 0.07^a^ (n = 13)	5.22 ± 0.42^a^ (n = 7)
Line A	57.9	39.9	27.4 ± 0.4^bc^ (n = 19)	29.8 ± 0.9^cd^ (n = 11)	0.85 ± 0.06^bc^ (n = 19)	2.87 ± 0.33^b^ (n = 11)
Line B	92.5	57.2	27.2 ± 0.5^bc^ (n = 11)	31.3 ± 0.8^bcd^ (n = 14)	1.16 ± 0.07^a^ (n = 11)	2.74 ± 0.30^b^ (n = 14)
Line C	45.1	30.7	26.9 ± 0.3^c^ (n = 29)	32.2 ± 0.6^b^ (n = 28)	0.99 ± 0.05^ab^ (n = 29)	2.70 ± 0.21^b^ (n = 28)
Line D	10.8	1.4	28.5 ± 0.3^a^ (n = 33)	31.7 ± 0.5^bc^ (n = 40)	0.95 ± 0.04^bc^ (n = 33)	2.49 ± 0.18^b^ (n = 40)
*ANOVA p-value*			0.0007	<0.0001	0.0003	<0.0001

The maximal TH interval retained is marked by the most proximal and distal primers with known B6 homozygous genotypes for a given donor region. The minimal TH interval retained is marked by the most proximal and distal primers with known TH homozygous genotypes for a given donor region. Fat pad weight is the sum of five fat pad weights (inguinal, epididymal, mesenteric, retroperitoneal including perirenal, and subscapular). Data are means ± SEM. Group means labeled with different letters are significantly different within a diet (*P* < 0.05)

We first compared subcongenic lines A and B that overlap and together cover the largest subcongenic donor region. Under chow feeding, the mean value of fat pad weight in line A was not statistically different from that in B6-homozygous control mice, while line B had significantly greater fat pad weight than controls, comparable to that in B6.TH-*tabw2* congenic mice (Table 1). These results suggest that the *tabw2* candidate region is most likely located within the unique segment retained in line B, extending distally from the marker *D6Mit29* (Figure 1). Interestingly, lines C and D, two nested subcongenic lines of the line B, but not overlapping each other, exhibited fat pad weights numerically intermediate between B6-homozygous control and B6.TH-*tabw2* congenic mice (Table 1). This may suggest that the effect of *tabw2* on obesity could be attributed to at least two subloci, named *tabw2a* (associated with line C) and *tabw2b* (associated with line D).

Under HFS diet feeding, on the other hand, all the subcongenic lines had significantly greater fat pad weight than B6-homozygous control mice, without genotype differences among the lines (Table 1). However, none of the subcongenic lines had fat pad weight that was as great as B6.TH-*tabw2* congenic mice.

### Indirect calorimetry, locomotor activity, and food intake

In order to elucidate metabolic mechanisms driving increased fat mass in the obesity mediated by *tabw2*, B6.TH-*tabw2* congenic, subcongenic, and B6-homozygous control mice fed either chow or HFS diets were allowed to acclimate to Comprehensive Laboratory Animal Monitoring System (CLAMS) for 24 hours followed by 48-hours continuous monitoring of heat production, respiratory exchange ratio (RER), locomotor activity, and food intake. The 2-day averages are presented in Table 2. Since body composition of *tabw2* mice varies mainly in adipose tissue [11], which exerts a limited contribution to total energy expenditure [15,16], CLAMS data were calculated per animal rather than correcting for body weight.

**Table 2:** **Energy expenditure (heat production), respiratory exchange ratio (RER), locomotor activity (ambulatory count/day), and food intake of B6.TH-*tabw2 *congenic, subcongenic, and B6-homozygous control mice on chow and HFS diets over a 24 hour period (males)**

**Group**	**Heat production (kcal/hour)**	**RER**	**Ambulatory count/day**	**Food intake (kcal/day)**
***Chow***				
Control (n = 10)	0.4879 ± 0.0117^ab^	0.918 ± 0.007^a^	19766 ± 1668^a^	n.d.
*tabw2* (n = 4)	0.4880 ± 0.0185^abc^	0.916 ± 0.012^a^	15163 ± 2638^ab^	n.d.
Line A (n = 10)	0.4691 ± 0.0117^bc^	0.907 ± 0.007^a^	18182 ± 1668^ab^	n.d.
Line B (n = 8)	0.4479 ± 0.0131^c^	0.916 ± 0.008^a^	13994 ± 1865^b^	n.d.
Line C (n = 9)	0.4556 ± 0.0123^bc^	0.923 ± 0.008^a^	19173 ± 1758^a^	n.d.
Line D (n = 14)	0.5028 ± 0.0099^a^	0.925 ± 0.006^a^	18796 ± 1410^a^	n.d.
*ANOVA p-value*	0.0081	0.5653	0.1850	
***HFS diets***				
Control (n = 12)	0.5541 ± 0.0107^a^	0.899 ± 0.007^a^	24571 ± 1523^a^	15.06 ± 0.52^b^
*tabw2* (n = 3)	0.5713 ± 0.0214^a^	0.906 ± 0.014^a^	15962 ± 3046^bc^	18.99 ± 1.04^a^
Line A (n = 11)	0.5305 ± 0.0112^a^	0.866 ± 0.007^b^	14741 ± 1591^c^	12.87 ± 0.54^c^
Line B (n = 8)	0.5398 ± 0.0131^a^	0.909 ± 0.008^a^	16928 ± 1865^bc^	16.18 ± 0.63^b^
Line C (n = 18)	0.5377 ± 0.0087^a^	0.899 ± 0.006^a^	17188 ± 1243^bc^	15.61 ± 0.42^b^
Line D (n = 23)	0.5497 ± 0.0077^a^	0.890 ± 0.005^a^	19463 ± 1100^b^	15.20 ± 0.37^b^
*ANOVA p-value*	0.4033	0.0014	0.0004	<0.0001

Data are means ± SEM. Group means labeled with different letters are significantly different within a diet (*P* < 0.05).*ND* no data.

Energy expenditure, determined as heat production (kcal/hour), was not largely different across the groups of mice fed chow, with the lowest value in line B. When animals were fed HFS diets, energy expenditure elevated in all mice compared to chow feeding, but there was no genotype effect.

Fuel source preference, as RER, was not significantly different between groups of mice fed chow. When animals were placed on HFS diets, RER values were generally lower compared to chow, demonstrating that whole body substrate metabolism was shifted towards fat oxidation. Notably, subcongenic line A mice showed significantly lower value of RER than other groups on HFS diets; this may be related with the significantly lower food intake in this group.

Locomotor activity, determined by the ambulatory count over a 24-hour period, was not significantly different among the groups of mice fed chow, with the lowest value in line B. On HFS diets, however, B6.TH-*tabw2* congenic and subcongenic mice showed significantly reduced locomotor activity compared to B6-homozygous control mice.

Food intake, determined as kcal consumed/day, was significantly higher (~ 26%) in B6.TH-*tabw2* congenic mice compared to B6-homozygous control mice on HFS diets. However, none of the subcongenic lines showed differences compared to control mice, except line A showed significantly lower caloric intake than controls. Despite the isocaloric or hypocaloric intake, the fat mass of subcongenic mice was ~1.4 – 1.6-fold higher than B6-homozygous control mice, potentially suggesting higher energy retention efficiency in subcongenic mice than controls.

## Discussion

In the current study, we conducted subcongenic mapping to fine map the *tabw2* QTL on chromosome 6 captured in B6.TH-*tabw2* congenic mice. We also assessed metabolic components regulating the energy homeostasis of B6.TH-*tabw2* congenic, subcongenic, and B6-homozygous control mice.

Our findings suggest that there are possibly at least two subloci, named *tabw2a* and *tabw2b*, responsible for the *tabw2*-mediated obesity on chow. The maximal interval for *tabw2a* would be a 38.5-Mb region between *D6Mit29* and *D6Mit254*. This region contains multiple obesity QTLs previously mapped, including *Efw* (epididymal fat weight) identified in F2 mice (NSY x C3H/He) [17], *Bw18* (body weight QTL 18) in SMXA mice [18], *Pbwg8* (postnatal body weight growth 8) in backcross mice [F1(B6 x CAST) x CAST] [19], *Egrm* (early growth rate, maternal effect 2) in F2 mice (SM/J x LG/J) [20], *Pfat2* (predicted fat percentage 2) in F2 mice (B6 x DBA/2J) [21], *Obq14* (obesity QTL 14) in F2 mice (NZO x SM) [22], and *Obwq3* (obesity and body weight QTL 3) in F2 mice (SM/J x NZB/BINJ) [23]. *Tabw2b* resides within a maximal interval of 10.8-Mb. A couple of obesity QTLs were previously mapped in this region, including *Wta2* (weight adult 2) identified in F2 mice (LG/J x SM/J) [24] and *Bwtq10* (body weight QTL 10) in F2 mice (SM/J x NZB/BINJ [23]. The concurrence of obesity QTLs in these regions may suggest that candidate gene(s) for the *tabw2* may be involved in obesity in other mouse models.

The present data also demonstrate a potential genetic complexity underlying the diet-induced obesity associated with *tabw2*. Unlike shown in chow feeding, there was no net effect of *tabw2a* and *tabw2b* on the fat pad weight in subcongenic line B mice fed HFS diets. At this stage the nature of *tabw2* effect on diet-induced obesity is unknown: however, it could arise from complex interactions among multiple subloci and/or interactions between the subloci and other genes that interact but have no effect on their own. Potentially, these interactions may occur in a context-dependent manner [25] involving a certain environment, such as HFS diets. Several examples of this complex genetic architecture for obesity QTLs have previously been reported and include a QTL on mouse chromosome 7 [26] and *Wg2a-d* (weight gain in high growth mice 2a-d) [27] and *Pbwg1.1-8* (postnatal body weight growth1.1-8) [28] on mouse chromosome 2. Also, we cannot rule out the possibility that the end-proximal portion of the congenic interval, not captured in subcongenic lines in this study, might be required for the full congenic effect on diet-induced obesity. Previously, we performed fine mapping of the *tabw2* locus by standard linkage analysis using F2 mice fed HFS diets and gained only a rough map position of 15 cM [11], probably due to the genetic complexity underlying the diet-induced obesity in *tabw2* congenic mice. Nonetheless, the 15 cM interval overlaps the *tabw2a* interval.

Using CLAMS experiments, we report that modest, but significant, hypoactivity emerged in B6.TH-*tabw2* congenic and subcongenic mice when animals were placed on HFS diets. This modest decrease in activity, however, did not significantly change the energy expenditure as assessed by heat production calculated from VO_2_. Therefore, it appears that these VO_2_ measurements in indirect calorimetric system reflect relative energy expenditure rather than absolutely accurate measurements as previously discussed [29]. Similar observations of little effect of changes in locomotor activity on energy expenditure were previously reported in other indirect calorimetric studies using mice [30,31]. Overfeeding usually results in compensatory increases in energy expenditure and physical activity in an attempt to maintain constant body mass [32]. Therefore, an inability to increase locomotor activity in response to HFS diets over time could lead to the diet-induced obesity mediated by *tabw2,* although it does not fully explain the full congenic phenotype. Currently, the mechanism by which decreases in locomotor activity in the diet-induced obesity associated with *tabw2* is unknown. Possibilities include lack of motivation or the presence of physical limitations accompanied by increased body fat mass.

Unlike the reduced locomotor activity, increased food intake was only shown in B6.TH-*tabw2* congenic mice fed HFS diets. Therefore, we speculate that the increased food intake might be a contributing factor for the full congenic phenotype of adiposity. However, as food intake data are not presently available on chow, we cannot conclude whether the hyperphagia is constitutive or disrupted compensatory response to HFS diets.

## Conclusions

We have created subcongenic lines, with smaller TH donor segments from the *tabw2* congenic interval, which develop detectable phenotypes, thus narrowing target intervals for positional cloning. The data presented here also identify potential pathological mechanisms underlying the diet-induced obesity mediated by *tabw2*. Future studies to further define subloci of *tabw2* and functional studies involving candidate genes could provide useful knowledge in understanding genetic contributions in human obesity.

## Methods

### Animals and diets

All mice were allowed free access to food and water in a temperature and humidity controlled room with a 12-hour light/dark cycle. At 4 weeks of age, mice were weaned onto standard rodent chow (Purina 5001, PMI Nutrition, Brentwood, MO, USA) or HFS diets (32% kcal from fat and 25% kcal from sucrose) (12266B, Research Diets, New Brunswick, NJ, USA) and maintained. Detailed composition of chow and HFS diets are presented in Table 3. All animal studies were carried out with the approval of Marshall University Animal Care and Use Committee. Mice were euthanized by CO_2_ asphyxiation.

**Table 3:** Diet composition of chow and high fat-high sucrose diets (HFS)

			** Chow**			** HFS**		
			**gm%**	**kcal%**		**gm%**	**kcal%**	
Protein			23.9	28.5		18.5	16.8	
Carbohydrate			48.7	58.0		56.7	51.4	
Fat			5.0	13.5		15.6	31.8	
		Total		100.0			100.0	
		kcal/gm	3.36			4.41		
***Ingredient***								
	**Chow**	**HFS**		**Chow**	**HFS**		**Chow**	**HFS**
Arginine,%	1.41	0.55	Linolenic Acid,%	0.10	0.218	Zinc, ppm	79	32
Cystine,%	0.31	0.11	Other omega-3 Fatty Acids,%	0.19		Manganese, ppm	70	65
Glycine,%	1.21	0.27	Total Saturated Fatty Acids,%	1.56	4.02	Copper, ppm	13	6.5
Histidine,%	0.57	0.42	Total Monounsaturated Fatty Acids,%	1.60	4.04	Cobalt, ppm	0.9	
Isoleucine,%	1.14	0.69	Fiber (crude),%	5.1		Iodine, ppm	1	0.2
Leucine,%	1.83	1.44	Cellulose, BW200,%		2.8	Chromium, ppm	1.2	2.2
Lysine,%	1.41	1.20	Starch,%	31.9	20.6	Selenium, ppm	0.3	0.2
Methionine,%	0.67	0.79	Glucose,%	0.22	42	Carotene, ppm	2.3	
Phenylalanine,%	1.04	0.77	Fructose,%	0.3	14	Vitamin K, ppm	1.3	0.5
Tyrosine,%	0.71	0.83	Sucrose,%	3.7	28.8	Thiamin, ppm	16	6.3
Threonine,%	0.91	0.66	Lactose,%	2.01		Riboflavin, ppm	4.5	7.3
Tryptophan,%	0.29	0.19	Calcium,%	0.95	0.77	Niacin, ppm	120	31
Valine,%	1.17	0.85	Phosphorus,%	0.66	1.02	Pantothenic Acid, ppm	24	17
Serine,%	1.19	0.91	Phosphorus (non-phytate),%	0.39		Choline, ppm	2250	788
Aspartic Acid,%	2.81	1.11	Potassium,%	1.18	0.54	Folic Acid, ppm	7.1	2.1
Glutamic Acid,%	4.37	3.48	Magnesium,%	0.21	0.05	Pyridoxine, ppm	6	7.4
Alanine,%	1.43	0.46	Sulfur,%	0.36	0.04	Biotin, ppm	0.3	0.2
Proline,%	1.49	1.62	Sodium,%	0.4	0.35	B_12_, mcg/kg	50	10.4
Taurine,%	0.02		Chlorine,%	0.67	0.17	Vitamin A, IU/gm	15	4.2
Cholesterol, ppm	200		Fluorine, ppm	16		Vitamin D, IU/gm	4.5	1.0
Linoleic Acid,%	1.22	6.9	Iron, ppm	270	42	Vitamin E, IU/kg	42	52

### Generation of subcongenic lines of mice

B6.TH-*tabw2* congenic mice and B6-homozygous control mice were previously established [11]. First, a B6.TH-*tabw2* congenic strain was crossed with B6-homozygous control strain, and the resultant F1 hybrids were intercrossed to produce F2 progeny. The F2 mice were then genotyped with simple sequence length polymorphism (SSLP) primers spaced throughout the congenic interval to search for crossovers. When a desirable crossover was identified within the region of interest, the recombinants were backcrossed to B6-homozygous control strain to duplicate the fragment. Male and female heterozygous backcrossed mice were then intercrossed to derive progeny that are TH homozygous for the region of interest and B6 homozygous for the rest of the chromosome.

### Genotyping by PCR

Genomic DNA was extracted from tail tips using proteinase K [11] and two series of salt precipitation steps. The DNA was PCR amplified using SSLP primers synthesized (Sigma-Aldrich, St. Louis, MO, USA) based on sequences from Mouse Genome Informatics ( http://www.informatics.jax.org). The thermal cycle consisted of 95°C for 2 min, followed by 49 cycles of 94°C (20 sec), 50°C (30 sec) and 72°C (40 sec) and a final extension at 72°C (7 min). Amplified products were electrophoretically separated on 3% MetaPhor agarose (50184, Lonza, Rockland, ME, USA)/1% agarose (0710-500G, Amresco, Solon, OH, USA) gels in 0.5 x tris-borate-EDTA buffer, pH 7.4. The DNA was visualized by ethidium bromide (E-1510, Sigma-Aldrich) staining.

### Subcongenic mapping of the adiposity trait

For subcongenic mapping, B6.TH-*tabw2* congenic, sucongenic, and B6-homozygous control mice fed either chow or HFS diets were weighed and killed at 14–17 weeks of age (non-fasting). Five white fat pads (inguinal, epididymal, mesenteric, retroperitoneal including perirenal, and subscapular) were then collected and weighed. The sum of these five pad weights was used as the adiposity trait.

### Indirect calorimetry, locomotor activity, and food intake

At 14–15 weeks of age, food intake, energy expenditure, RER, and locomotor activity were measured using an eight-chamber CLAMS (Columbus Instruments, Columbus, OH, USA). In this system, total oxygen consumption (VO_2_) and carbon dioxide production (VCO_2_) were measured, and VO_2_ was converted to individual heat production (kcal/hour) by Columbus software. This software calculates the heat production by multiplying the calorific value $CV=3.815+\left(1.232\times RER\right)$ by the observed $VO_{2}\left(Heat=CV\times VO_{2}\right)$. RER is the ratio between the VCO_2_ and $VO_{2}\left(RER=VCO_{2}/VO_{2}\right)$, reflecting the daily whole-body oxidation rate of carbohydrate and fat; ~ 0.7 (complete reliance on fat oxidation) and 1.0 (complete reliance on carbohydrate oxidation). A system of infrared beams detects movement of animals in CLAMS, and locomotor activity was determined as ambulatory count, the number of times different beams were broken in either the x- or y-axes during an interval. The CLAMS allows continuous measurement of food intake by each animal. All mice were acclimatized to monitoring cages for 24 hours prior to an additional 48 hours of recordings under the regular 12-hour light–dark cycle.

### Statistical analysis

All response variables were analyzed using ANOVA (SAS version 9.3, Cary, NC), with group, diet, and interaction effects in the model. Differences among means were sliced by diet (group differences only compared within diets), and tested using Fisher's LSD at *P* < 0.05. All data are presented as least squares means ± SEM.

## Competing interests

The authors declare that they have no competing interests.

## Authors’ contributions

TPS and MNA constructed the subcongenic lines. DU and KDD did additional genotyping and KDD maintained the mouse colony. TPS phenotyped mice for mapping. TPS and XM did CLAMS experiments. AMS conducted statistical analysis of the data. JHK conceived the study and was primarily responsible for its coordination and design. AMS and JHK drafted the manuscript, tables and figures. All authors read and approved the final manuscript.
